# Body image perception and emotional awareness in adults with celiac disease: A cross-sectional study

**DOI:** 10.1097/MD.0000000000048431

**Published:** 2026-05-15

**Authors:** Kinga Kot, Agnieszka Dobrowolska, Iwona Krela-Kaźmierczak, Maciej Głowacki, Ewa Misterska

**Affiliations:** a Varsovia University of Business & Applied Sciences, Poznań Institute of Psychology and Pedagogy, Poznan, Greater Poland Voivodeship, Poland; b Poznan University of Medical Sciences, Department and Clinic of Gastroenterology, Dietetics and Internal Diseases, Poznan, Greater Poland Voivodeship, Poland; c Poznan University of Medical Sciences, Department of Pediatric Orthopaedics and Traumatology, Poznan, Greater Poland Voivodeship, Poland; d Poznan University of Medical Sciences, Department of Mental Health, Poznan, Greater Poland Voivodeship, Poland.

**Keywords:** alexithymia, BES, body image, celiac disease, gluten-free diet, TAS-26

## Abstract

We aimed to investigate the relationship between alexithymia (a trait characterized by difficulty identifying and describing emotions) and body image (the perception and evaluation of one’s own body) in adults with celiac disease (CD) compared to healthy controls. A cross-sectional study included 96 adults with CD (diagnosed via laboratory tests, endoscopy, or clinical assessment) and 96 healthy controls. Alexithymia, defined as difficulty identifying and expressing emotions, was evaluated alongside body image to assess its clinical relevance in adults with CD. All participants completed the Polish versions of the Toronto Alexithymia Scale-26 and Body Esteem Scale. CD patients showed higher total Toronto Alexithymia Scale-26 scores (indicating greater severity of alexithymic traits) (*P* = .006), particularly in difficulty identifying feelings and reduced daydreaming domains (*P* = .005 for both). Alexithymia was diagnosed in 26% of CD patients versus 8% of controls (*P* < .001). Male CD patients scored lower than healthy controls on Body Esteem Scale domains of physical attractiveness, body strength, and physical condition (*P* < .001, *P* = .010, *P* < .001, respectively). Alexithymia and body image disturbances are more pronounced in male CD patients than in healthy controls. Difficulty identifying feelings negatively affects body perception, highlighting the importance of psychosocial assessment in the management of CD. Addressing emotional awareness and body image could improve clinical care and quality of life in adults with CD.

## 1. Introduction

Celiac disease (CD) is an autoimmune disorder characterized by small intestinal mucosal damage triggered by an immune response to gluten peptides, with clinical improvement following a strict gluten-free diet (GFD).^[1,2]^ CD affects approximately 0.5 to 1% of the general population, with higher prevalence among first-degree relatives of affected individuals,^[3–5]^ and nonclassical forms with extraintestinal manifestations are increasingly recognized. Adults with CD may present with anemia, fatigue, headache, neurological symptoms, and psychiatric disorders, whereas children often exhibit short stature, growth retardation, and fatigue.^[1,6,7]^ Neurological manifestations, though rare in children, may affect up to 36% of adult patients at presentation.^[1]^

Psychiatric comorbidities affect around 10% of CD patients and commonly include depression, anxiety, eating disorders, and alexithymia.^[8–11]^ Alexithymia, defined as difficulty identifying and expressing emotions, has been linked to various gastrointestinal and autoimmune disorders and is conceptualized as a disorder of emotion regulation, involving impaired recognition of internal states and increased focus on external events.^[12,13]^ The first attempts to verify the hypotheses about the relationship between alexithymia and CD as well as gluten intolerance were made over the last decade.^[9,10]^ Collin et al^[9]^ did not confirm a relationship between an increased level of alexithymia, diagnosis of CD and adherence to a GFD. However, Kreitler and Kreitler^[10]^ indicated a higher level of alexithymia in the CD patient group compared to the healthy control group. By increasing the risk of depressive disorders, alexithymia may contribute to psychosocial burden in CD and negatively influence both GFD adherence and quality of life.^[8,14–17]^

Body image, defined as perception, evaluation, and satisfaction with one’s own body, is another important psychosocial factor in CD, often negatively affected by gastrointestinal symptoms, nutritional deficiencies, and comorbid mood disorders.^[14–18]^ Impaired body image may contribute to psychological distress and further exacerbate emotional dysregulation, creating a cycle that impacts patient well-being and treatment outcomes.

Given these considerations and the lack of conclusive evidence regarding the relationship between alexithymia and body image in CD, we aimed to investigate the association between body image and alexithymia in adult males and females with CD compared to healthy controls. Understanding these psychosocial aspects may inform more comprehensive clinical management and improve patient outcomes in CD.

## 2. Methods

### 2.1. Study design

Adhering to the Strengthening the Reporting of Observational Studies in Epidemiology guidelines for observational studies, this research was conducted as a cross-sectional comparison between 2 groups: patients treated for CD (n = 96) and healthy controls (n = 96). The study was carried out from May 2018 to September 2021. All clinical and sociodemographic data were collected, and questionnaires were administered during routine patient visits. The investigator (Kinga Kot) was available throughout the visits to provide explanations or clarifications if required by the participants.

### 2.2. Analyzed data

In both study groups, the analysis included sociodemographic variables such as age, sex, place of residence, marital status, education, and occupational activity. For CD patients, additional information was collected, including age at diagnosis, time from first symptoms to diagnosis, diagnostic methods, family history of CD, comorbidities, and anthropometric measures (weight and height).

Furthermore, the following variables were assessed using a 10-point scale: severity and duration of CD symptoms after gluten consumption, adherence to a GFD, frequency of intentional gluten intake, and the perceived impact of the GFD on daily life, including social activities, family meal preparation, and professional functioning. The effects of CD symptoms and GFD requirements on both professional life and emotional well-being were also evaluated.

### 2.3. Inclusion and Exclusion Criteria

Participants were selected deliberately according to predefined inclusion and exclusion criteria. For the clinical group, inclusion criteria were: age 18 to 65 years; male or female gender; diagnosis of CD confirmed by laboratory tests, endoscopic and histopathological examination, or by physician interview and symptom assessment after gluten consumption; and provision of written informed consent. Patients with intellectual disabilities were excluded.

For the healthy control group, inclusion criteria were: age 18 to 65 years; male or female gender; absence of a CD diagnosis (confirmed by laboratory tests, endoscopic and histopathological examination, or physician interview and symptom assessment after gluten consumption); and written informed consent. Healthy controls with intellectual disabilities were excluded from the study.

### 2.4. Sample size estimation

The required sample size was estimated using the formula for a finite population: n = N/1 + (4d^2^ (N−1))/uα^2^), where n is the required number of participants, N is the population of individuals with CD and food intolerance in Poland (400,000),^[19]^ uα = 0.05 represents the significance level, and d = 10% is the acceptable margin of error. Based on these calculations, the minimum sample size for participants with CD was determined to be n = 96. The control group was matched with the same number of participants (n = 96).

**Figure 1.**
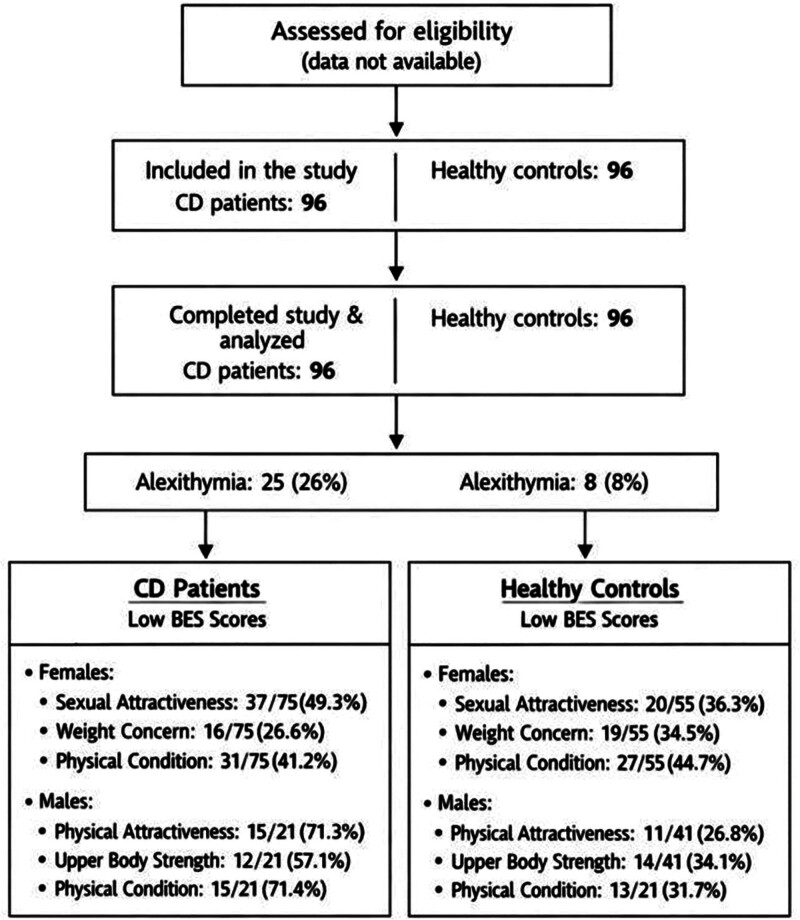
Flow of participants through the study with BES and TAS-26. Percentages reflect the proportion of patients with high TAS-26 and low BES scores in each subgroup (females and males). CD = celiac disease, BES = body esteem scale, TAS-26 = Toronto Alexythymia Scale-26.

The flow of participants through each stage of the study is illustrated in Figure 1.

### 2.5. Ethical issues

The study was approved by the Bioethics Committee (No. 527/2018) and was conducted in accordance with the ethical standards of the 1964 Declaration of Helsinki and its subsequent amendments. All participants received detailed information about the study’s objectives and were assured of confidentiality before providing their written informed consent.

### 2.6. Study measures

Standardized psychological instruments were applied. Alexithymia levels and dimensions were assessed using the Polish version of the Toronto Alexithymia Scale-26 (TAS-26),^[12,20]^ while body image and its domains were evaluated with the Polish version of the Body Esteem Scale (BES).^[21,22]^

The TAS-26 is a widely used, reliable, and validated self-report measure of alexithymia. It consists of 26 items rated on a 5-point Likert scale, with responses reflecting the degree to which each statement applies to the respondent. The scale is designed to assess core alexithymic traits related to emotional awareness and emotional processing. Items are grouped into 4 factors: difficulty identifying and distinguishing feelings from bodily sensations (factor 1), difficulty describing feelings (factor 2), reduced daydreaming (factor 3), and externally oriented thinking (factor 4). Higher total scores indicate greater severity of alexithymic characteristics. The total score ranges from 26 to 130 points. In the present study, we used the Polish adapted version of the TAS-26, which has demonstrated satisfactory psychometric properties in Polish samples. Clinically established cutoff values proposed by the Toronto group were applied: scores ≥ 78 indicate alexithymia, whereas scores ≤ 64 indicate no alexithymia.^[12,20]^

The BES Polish adaptation^[22]^ is comprised of 35 items grouped into 3 gender-specific subscales. The subscales for women include sexual attractiveness, weight concern, and physical condition, whereas for men body esteem is assessed through Physical Attractiveness, Upper Body Strength, and Physical Condition. Each BES item is scored on a 5-point Likert-type scale, where 1 corresponds to strongly negative feelings, 5 to strongly positive feelings, and 3 represents a neutral midpoint. Higher scores indicate more positive body esteem. The Polish adaptation has demonstrated high reliability and validity.^[21,22]^ For this study, both total and subscale scores were analyzed to examine gender-specific differences in body image between adults with CD and healthy controls.

### 2.7. Statistical analysis

For quantitative variables, we calculated the mean, 95% confidence intervals, range, and standard deviations (SDs). For qualitative variables, frequencies and percentages of observations in each category were reported. The distribution of continuous variables was assessed using the Shapiro–Wilk test. Differences between 2 groups were evaluated with the Mann–Whitney U test, while comparisons among multiple groups were performed using the Kruskal-Wallis test followed by the Conover-Iman post hoc test.

Spearman rank correlation coefficients (*r*_S_) were used to assess relationships between quantitative variables. Associations between categorical variables were examined using the chi-square test of independence or the Fisher-Freeman-Halton test when appropriate. A *P* value < .05 was considered statistically significant. All analyses were performed using Statistica 13 (TIBCO Software Inc., Palo Alto, CA) and PQStat (PQStat Software, Poznań, Greater Poland Voivodeship, Poland).

To reduce potential bias, strict inclusion/exclusion criteria were applied, and all participants completed standardized questionnaires (TAS-26, BES) under the supervision of the same investigator. Sociodemographic variables were recorded and considered in analyses to account for group differences.

## 3. Results

### 3.1. Study samples characteristics

Table 1 presents detailed sociodemographic characteristics of study groups. Females constituted 78.1% of CD patients. Mean age of CD patients was 39.7 (14.2) years and of healthy controls 40.0 (14.5) years (*P* = .80).

**Table 1:** Study samples characteristics: sociodemographic data.

	CD patients		Healthy controls		
	n	%	N	%	*P* value
Sex					
Females	75	78.1	55	57.3	**< .001**
Males	21	21.9	41	42.7	
Educational level					
Elementary	1	1.0	1	1.0	**.009**
Vocational	3	3.1	15	15.6	
Secondary	36	37.5	40	41.7	
University	54	56.3	39	40.6	
Marital status					
Single	30	31.3	32	33.3	**.03**
Married	59	61.5	44	45.8	
Widowed	3	3.1	7	7.3	
Divorced	4	4.2	13	13.5	
Place of residence					
Rural	12	12.5	12	12.5	.08
City below 25,000 inhabitants	13	13.5	27	28.1	
City between 25,000 and 200,000 inhabitants	18	18.8	17	17.7	
City over 200,000 inhabitants	53	55.2	40	41.7	
Employment status					
Employed: full/part time	75	78.1	76	79.2	** .002**
Retired	4	4.2	7	7.3	
Receiving a disability pension	11	11.5	0	0	
Unemployed	5	5.2	12	12.5	
Students	1	1.0	1	1.0	
	Mean (SD)	Min–Max	Mean (SD)	Min–Max	*P* value
Age at present [yrs]	39.7 (14.2)	18–65	40.0 (14.5)	19–65	.80
Working time per week [h]	30.3 (19.8)	0–75	32.9 (19.0)	0–70	.19

CD = celiac disease, Max = maximum value, Min = minimum value, n/N = number of patients, N% = percentage of respondents.

*P* values showing statistically significant difference are written in bold.

The differences between the 2 study groups are significant in regards to sex (*P* < .001), educational level (*P* = .009), marital (*P* = .03) and employment status (*P* = .002).

### 3.2. Clinical data and data regarding adherence to a GFD in CD sample

The mean age at diagnosis in the CD group was 33.6 (13.4) years, and the time from onset of symptoms to diagnosis was on average 5.4 (5.1) years. The diagnosis of CD was made in patients based on laboratory tests in 39.6% of patients, and on endoscopy in 51% patients. In addition, 25% of the surveyed patients reported the diagnosis of CD in a family member, 54.2% also stated CD comorbidities, 27.1% of whom indicated autoimmune diseases. Detailed data is presented in Table 2.

**Table 2:** Clinical characteristics in the group of patients with CD.

	Mean (SD)	Min–Max	Median
Age at diagnosis [yrs]	33.6 (13.4)	1–70	32
Duration of symptoms prior to diagnosis [yrs]	5.4 (5.1)	0.5–24	4
Height [cm]	166.1 (7.8)	154–188	166
Weight [kg]	57.8 (9.7)	41–92	56
	n	%	
Method of diagnosis of CD			
Laboratory test	38	39.6	
Endoscopy and histopathology	49	51.0	
Medical interview and symptom analysis	2	2.1	
Other	6	6.3	
Diagnosis of CD in family members	24	25	
Comorbidities	44	45.8	
Including ADs	26	27.1	

AD = autoimmune disease, CD = celiac disease, Max = maximum value, Min = minimum value, N% = percentage of respondents, SD = standard devation.

*P* values showing statistically significant difference are written in bold.

Data regarding intensity of CD symptoms and compliance to a GFD are summarized in Table 3. Subjects with a high score, an average of 7.2 (SD 2.8) on a 10-point scale, assessed the degree of adherence to a GFD, where the severity of CD symptoms was estimated at 5.3 (SD 2.7), the assessment of continuing symptoms following gluten exposure was rated at 4.4 (SD 2.5). Detailed data on the impact and exacerbation of CD symptoms on hospitalizations and emotional burden, as well as dietary requirements, are also included in Table 3.

**Table 3:** Data regarding adherence to a GFD in the group of patients with CD.

	Mean (SD)	Min–Max	Median
Severity of CD symptoms[1–10]*	5.3 (2.7)	1–10	5
Assessment of continuing CD symptoms following gluten exposure[1–10]*	4.4 (2.5)	1–10	4
Evaluation of degree of adherence to a GFD[1–10]*	7.2 (2.8)	1–10	8
Evaluation of conscious gluten consumption[1–10]†	3.3 (2.4)	1–10	3
Impact of GFD on social activity[1–10]‡	4.6 (2.0)	1–10	5
Impact of GFD on family life[1–10]‡	4.1 (2.2)	1–10	5
Impact of GFD on professional life[1–10]‡	4.6 (2.0)	1–10	5
Impact and exacerbation of CD symptoms on hospitalization[1–10]‡	4.1 (2.5)	1–10	4
Impact of CD symptoms on emotional burden[1–10]‡	3.4 (2.2)	1–9	3

CD = celiac disease, GFD = gluten-free diet, SD = standard deviation.

* 1–10, where 1 means: slight severity of symptoms/continuation of symptoms/degree of adherence to diet, 5 means: moderate degree of severity of symptoms/ continuation of symptoms/degree of adherence to diet and 10 means: very high degree of severity of symptoms, continuation of symptoms/ degree of adherence to the diet.

† 1–10, where 1 means never, 5 means: once every few weeks, and 10 means: at least once a week.

‡ 1–10, where 1 means: diet requirements/celiac symptoms have a slight negative impact, 5 means: moderate negative impact, and 10 means: very negative impact.

### 3.3. Outcome measures-scores distribution

Detailed results regarding the average TAS-26 and BES scores obtained by the subjects are presented in Table 4. The average total score of TAS-26 in the CD group was 69.7 (SD 11.5), and in the control group 64.8 (SD 11.1). The groups differ in terms of: total TAS-26 score (*P* = .006), domains: difficulty in identifying feelings and reduced daydreaming (*P* = .005 and *P* = .005), as well as the frequency of alexithymia diagnosis (26% in the CD group and 8% in the control group, the difference is significant at *P* < .001).

**Table 4:** Distribution of scores within TAS-26 and BES in both study groups.

	CD patients			Healthy controls			*P* value
TAS-26	Mean (SD)	95% CI (from–to)	Range (Min–Max)	Mean (SD)	95% CI (from–to)	Range (Min–Max)	
Difficulty in identifying feelings	28.9 (7.3)	27.4–30.4	15–44	26.0 (7.5)	24.5–27.5	15–43	** .005**
Reduced daydreaming	13.7 (4.2)	12.9–14.6	5–22	12.1 (3.8)	11.3–12.8	5–21	** .005**
Externally oriented thinking	14.1 (2.6)	13.6–14.6	9–21	13.6 (2.3)	13.1–14.1	9–20	.08
Difficulty in describing feelings	13.0 (3.0)	12.4–13.6	5–20	13.1 (3.9)	12.3–13.9	5–21	.95
Total score	69.7 (11.5)	67.3–72.0	40–95	64.8 (11.1)	62.5–67.1	44–90	** .006**
% of patients above the cutoff level (78 of 130 pts)	26			8			** < .001**
BES for females							
Sexual Attractiveness	46.8 (8.0)	45.0–48.6	34–65	48.3 (9.5)	45.7–50.8	27–65	.22
Weight Concern	33.4 (8.2)	31.5–35.3	11–50	32.3 (9.4)	29.8–34.9	13–50	.56
Physical Condition	31.1 (7.3)	29.4–32.7	17–45	30.8 (8.1)	28.6–33.0	16–45	.68
BES for males							
Physical Attractiveness	36.0 (6.8)	32.9–39.1	22–55	44.4 (8.4)	41.7–47.0	27–55	** < .001**
Upper Body Strength	28.7 (7.4)	25.3–32.1	15–45	34.6 (8.3)	32.0–37.2	16–45	** .009**
Physical Condition	38.8 (10.7)	34.0–43.7	21–62	49.5 (11.6)	45.8–53.2	23–65	** < .001**

BES = Body Esteem Scale, CD = celiac disease, CI = confidence interval, GFD = gluten-free diet, Max = maximum value, Min = minimum value, SD = standard deviation, TAS-26 = Toronto Alexithymia Scale-26.

*P* values showing statistically significant difference are written in bold.

For the BES subscales in women (sexual attractiveness, weight control, and physical condition) there were no statistically significant differences between the groups. Thanks to the use of the sten scale, it was shown that in the sexual attractiveness subscale, the most frequently observed values were low stens in CD patients (49.3%) or low and high in healthy controls (36.3% and 36.3%, respectively), in the weight concern subscale medium stens were most common in CD patients (47.9%), followed by low or high stens in healthy controls (34.5% and 34.5% respectively). In the subscale of physical condition, the most common values in both study groups were low stens (41.2% and 44.7%, respectively). Detailed results are presented in Table 5.

**Table 5:** BES results in the group of women after reference to sten norms in both study groups.

BES for females				
Level	Low	Medium	High	*P* value
Sexual Attractiveness				
CD patients	37	22	16	.28
	49.3	29.3	21.2	
Healthy controls	20	15	20	
	36.3	27.4	36.3	
Weight concern				
CD patients	16	36	23	.46
	26.6	47.9	30.5	
Healthy controls	19	17	19	
	34.5	31	34.5	
Physical Condition				
CD patients	31	16	28	.54
	41.2	21.3	37.2	
Healthy controls	27	12	16	
	44.7	21.8	28.9	
BES for males				
Physical Attractiveness				
CD patients	15	4	2	** < .001**
	71.3	19	9.5	
Healthy controls	11	6	24	
	26.8	14.7	58.5	
Upper Body Strength				
CD patients	12	5	4	** .010**
	57.1	23.8	19.1	
Healthy controls	14	9	18	
	34.1	22	43.9	
Physical Condition				
CD patients	15	4	2	** < .001**

BES = Body Esteem Scale, CD = celiac disease.

*P* values showing statistically significant difference are written in bold.

Regarding the BES subscales for men: physical attractiveness, body strength and physical condition, patients with CD obtained significantly lower results compared to the control group (differences are statistically significant at the levels of *P* < .001, *P* = .01 and *P* < .001). Thanks to the use of the sten scale, it was shown that among patients with CD in each of the subscales, the most frequently observed values were low stens: in the subscale of physical attractiveness at the level of 71.3%, in the subscale of body strength at the level of 57.1%, and in the subscale of physical condition at the level of 71.4%. It is worth noting that men from the control group obtained high stens most frequently, both in the subscales of physical attractiveness (58.5%), physical condition (46.4%) and body strength (43.9%). Detailed results are presented in Table 5. Figure 1 illustrates participant distribution, including the number of individuals with alexithymia (as indicated by TAS scores ≥ 78, based on clinically established cutoff values) and disturbed body image (as indicated by low BES scores).

### 3.4. Analysis by sociodemographic and clinical data

Details regarding relation between TAS-26, BES and sociodemographic and clinical data can be seen in Table 6 and Table 7.

**Table 6:** Correlation between sociodemographic variables and TAS-26 and BES in the group of patients with CD.

	CD patients				
	Sex	Age at present	Marital status	Place of residence	Employment status
	*P* value	*r*_S_, *P* value	*P* value	*P* value	*P* value
TAS-26					
Difficulty in identifying feelings	.46	*r*_S_ = -0.10 *P* = .34	.19	.38	.99
Reduced Daydreaming	.26	*r*_S_ = 0.11 *P* = .29	.11	.17	** .035**
Externally oriented thinking	.46	*r*_S_ = 0.02 *P* = 0,86	.24	** .04**	.61
Difficulty in describing feelings	.40	*r*_S_ = -0.07 *P* = .51	.06	.08	.66
Total score	.88	*r*_S_ = -0.05 *P* = .64	.28	.57	.34
BES for females					
Sexual Attractiveness	No data available	*r*_S_ = 0.03 *P* = .77	.52	.17	.17
Weight Concern	No data available	*r*_S_ = 0.04 *P* = .73	.23	.85	.07
Physical Condition	No data available	*r*_S_ = 0.02 *P* = .874	.42	.31	.13
BES for males					
Physical Attractiveness	No data available	*P* = .10	No data available	.68	.79
Upper Body Strength	No data available	*P* = .08	No data available	.75	.73
Physical Condition	No data available	*P* = .36	No data available	.41	.82

BES = Body Esteem Scale, CD = celiac disease, *r*_S_ = Spearman rank correlation coefficient, TAS-26 = Toronto Alexithymia Scale-26.

*P* values showing statistically significant difference are written in bold.

**Table 7:** Correlation between working time, age at the time of diagnosis, duration of symptoms before diagnosis, and TAS-26 and BES in the group of patients with CD.

	CD patients			
	Working time per week	Age at diagnosis	Duration of symptoms prior to diagnosis	Comorbidities
	*r*_S_, *P* value	*r*_S_, *P* value	*r*_S_, *P* value	*P* value
TAS-26				
Difficulty in identifying feelings	*r*_S_ = -0.09 *P* = .41	*r*_S_ = -0.08 *P* = .42	*r*_S_ = 0.01 *P* = .91	.97
Reduced Daydreaming	*r*_S_ = 0.16 *P* = .11	*r*_S_ = 0.12 *P* = .25	*r*_S_ = -0.13 *P* = .19	** .026**
Externally oriented thinking	*r*_S_ = 0.05 *P* = .62	*r*_S_ = 0.07 *P* = .50	*r*_S_ = -0.11 *P* = .29	.39
Difficulty in describing feelings	*r*_S_ = -0.09 *P* = .43	*r*_S_ = -0.02 *P* = .88	*r*_S_ = -0.06 *P* = .59	.19
Total score	*r*_S_ = 0.01 *P* = .97	*r*_S_ = 0.01 *P* = .98	*r*_S_ = -0.12 *P* = .26	.45
BES for females				
Sexual Attractiveness	*r*_S_ = -0.09 *P* = .43	*r*_S_ = 0.07 *P* = .58	*r*_S_ = 0.04 *P* = .76	.74
Weight Concern	*r*_S_ = -0.11 *P* = .31	*r*_S_ = 0.06 *P* = .63	*r*_S_ = 0.01 *P* = .92	.93
Physical Condition	*r*_S_ = -0.04 *P* = .72	*r*_S_ = 0.01 *P* = .92	*r*_S_ = 0.10 *P* = .35	.68
BES for males				
Physical Attractiveness	*r*_S_ = 0.23 *P* = .32	*r*_S_ = -0.29 *P* = .21	*r*_S_ = -0.14 *P* = .54	1.00
Upper Body Strength	*r*_S_ = 0.24 *P* = .29	*r*_S_ = -0.30 *P* = .19	*r*_S_ = -0.15 *P* = .50	.91
Physical Condition	*r*_S_ = 0.19 *P* = .41	*r*_S_ = -0.21 *P* = .37	*r*_S_ = -0.32 *P* = .15	.65

BES = Body Esteem Scale, CD = celiac disease, *r*_S_ = Spearman rank correlation coefficient, TAS-26 = Toronto Alexithymia Scale-26.

*P* value showing statistically significant difference is written in bold.

The relationship between the variables associated with CD symptoms and adherence to a GFD, and the results of TAS-26 and BES in the group of patients with CD are presented in Table 8, Table 9 and Table 10.

**Table 10:** Relationships between TAS-26 and BES in the group of patients with CD.

TAS-26	BES for females		
	Sexual Attractiveness	Weight Concern	Physical Condition
	*r*_S_, *P* value	*r*_S_, *P* value	*r*_S_, *P* value
Difficulty in identifying feelings	*r*_S_ = −0.03 *P* = .84	*r*_S_ = −0.08 *P* = .47	*r*_S_ = −0.18 *P* = .13
Reduced Daydreaming	*r*_S_ = −0.15 *P* = .21	*r*_S_ = 0.18 *P* = .12	*r*_S_ = −0.27 ***P* = .02**
Externally oriented thinking	*r*_S_ = 0.09 *P* = .45	*r*_S_ = 0.06 *P* = .61	*r*_S_ = 0.08 *P* = .47
Difficulty in describing feelings	*r*_S_ = −0.13 *P* = .27	*r*_S_ = −0.22 *P* = .06	*r*_S_ = −0.18 *P* = .12
Total score	*r*_S_ = −0.06 *P* = .59	*r*_S_ = −0.14 *P* = .22	*r*_S_ = −0.23 ***P* = .04**
	BES for males		
TAS-26	Physical Attractiveness	Upper Body Strength	Physical Condition
Difficulty in identifying feelings	*r*_S_ = −0.51 ***P* = .02**	*r*_S_ = −0.44 ***P* = .046**	*r*_S_ = −0.63 ***P* = .002**
Reduced daydreaming	*r*_S_ = 0.07 *P* = .47	*r*_S_ = 0.09 *P* = .71	*r*_S_ = 0.03 *P* = .89
Externally oriented thinking	*r*_S_ = −0.35 *P* = .13	*r*_S_ = −0.33 *P* = .11	*r*_S_ = −0.47 ***P* = .03**
Difficulty in describing feelings	*r*_S_ = 0.27 *P* = .24	*r*_S_ = 0.36 *P* = .10	*r*_S_ = 0.05 *P* = .84
Total score	*r*_S_ = −0.26 *P* = .24	*r*_S_ = −0.23 *P* = .32	*r*_S_ = −0.49 ***P* = .03**

BES = Body Esteem Scale, CD = celiac disease, *r*_S_ = Spearman rank correlation coefficient, TAS-26 = Toronto Alexithymia Scale-26.

*P* values showing statistically significant difference are written in bold.

**Table 8:** Relationships between variables related to CD symptoms and adherence to GFD, and TAS-26 and BES in the group of patients with CD.

	Severity of CD symptoms	Assessment of continuing CD symptoms following exposure to gluten	Assessment of degree of adherence to a GFD	Impact of CD symptoms on hospitalizations	Assessment of conscious gluten consumption
	*r*_S_, *P* value	*r*_S_, *P* value	*r*_S_, *P* value	*r*_S_, *P* value	*r*_S_, *P* value
TAS-26					
Difficulty in identifying feelings	*r*_S_ = −0.04 *P* = .73	*r*_S_ = −0.15 *P* = .15	*r*_S_ = −0.18 *P* = .08	*r*_S_ = 0.10 *P* = .31	*r*_S_ = 0.15 *P* = .13
Reduced daydreaming	*r*_S_ = 0.11 *P* = .26	*r*_S_ = 0.04 *P* = .69	*r*_S_ = −0.04 *P* = .73	*r*_S_ = 0.10 *P* = .35	*r*_S_ = 0.16 *P* = .13
Externally oriented thinking	*r*_S_ = 0.02 *P* = .81	*r*_S_ = 0.00 *P* = .97	*r*_S_ = 0.01 *P* = .99	*r*_S_ = −0.03 *P* = .78	*r*_S_ = 0.11 *P* = .30
Difficulty in describing feelings	*r*_S_ = −0.07 *P* = .52	*r*_S_ = −0.00 *P* = .98	*r*_S_ = −0.04 *P* = .73	*r*_S_ = 0.04 *P* = .67	*r*_S_ = 0.05 *P* = .66
Total score	*r*_S_ = 0.01 *P* = .78	*r*_S_ = −0.09 *P* = .40	*r*_S_ = −0.07 *P* = .52	*r*_S_ = 0.08 *P* = .45	*r*_S_ = 0.16 *P* = .12
BES for females					
Sexual Attractiveness	*r*_S_ = −0.07 *P* = .55	*r*_S_ = −0.16 *P* = .17	*r*_S_ = 0.10 *P* = .38	*r*_S_ = −0.31 ***P* = .007**	*r*_S_ = −0.05 *P* = .66
Weight Concern	*r*_S_ = −0.08 *P* = .48	*r*_S_ = −0.11 *P* = .37	*r*_S_ = 0.07 *P* = .54	*r*_S_ = −0.23 ***P* = .04**	*r*_S_ = −0.05 *P* = .69
Physical Condition	*r*_S_ = −0.05 *P* = .65	*r*_S_ = −0.06 *P* = .60	*r*_S_ = 0.13 *P* = .28	*r*_S_ = −0.41 ***P* < .001**	*r*_S_ = −0.04 *P* = .75
BES for males					
Physical Attractiveness	*r*_S_ = 0.21 *P* = .37	*r*_S_ = −0.11 *P* = .64	*r*_S_ = −0.13 *P* = .58	*r*_S_ = −0.19 *P* = .42	*r*_S_ = 0,37 *P* = .09
Upper Body Strength	*r*_S_ = 0.19 *P* = .41	*r*_S_ = −0.03 *P* = .89	*r*_S_ = −0.11 *P* = .630	*r*_S_ = −0.18 *P* = .43	*r*_S_ = 0.28 *P* = .21
Physical Condition	*r*_S_ = 0.21 *P* = .36	*r*_S_ = −0.19 *P* = .42	*r*_S_ = −0.14 *P* = .55	*r*_S_ = −0.32 *P* = .16	*r*_S_ = 0.12 *P* = .60

BES = Body Esteem Scale, CD = celiac disease, GFD = gluten-free diet, *r*_S_ = Spearman rank correlation coefficient, TAS-26 = Toronto Alexithymia Scale-26.

*P* values showing statistically significant difference are written in bold.

**Table 9:** Relationships between the impact of dietary requirements and symptoms of celiac disease on the functioning of patients, and TAS-26 and BES in the group of patients with CD.

	Impact of GFD on social activity	Impact of GFD on family life	Impact of GFD on professional life	Impact CD symptoms on emotional burden
	*r*_S_, *P* value	*r*_S_, *P* value	*r*_S_, *P* value	*r*_S_, *P* value
TAS-26				
Difficulty in identifying feelings	*r*_S_ = 0.14 *P* = .18	*r*_S_ = 0.22 ***P* = .03**	*r*_S_ = 0.03 *P* = .80	*r*_S_ = 0.10 *P* = .34
Reduced Daydreaming	*r*_S_ = 0.10 *P* = .33	*r*_S_ = 0.07 *P* = .50	*r*_S_ = 0.05 *P* = .61	*r*_S_ = 0.12 *P* = .91
Externally oriented thinking	*r*_S_ = 0.12 *P* = .24	*r*_S_ = −0.04 *P* = .68	*r*_S_ = 0.03 *P* = .76	*r*_S_ = −0.68 *P* = .50
Difficulty in describing feelings	*r*_S_ = −0.05 *P* = .61	*r*_S_ = 0.06 *P* = .54	*r*_S_ = 0.11 *P* = .29	*r*_S_ = 0.08 *P* = .47
Total score	*r*_S_ = 0.15 *P* = .13	*r*_S_ = 0.16 *P* = .12	*r*_S_ = 0.05 *P* = .65	*r*_S_ = 0.03 *P* = .74
BES for females				
Sexual Attractiveness	*r*_S_ = 0.01 *P* = .96	*r*_S_ = −0.15 *P* = .21	*r*_S_ = −0.20 *P* = .09	*r*_S_ = −0.20 *P* = .08
Weight Concern	*r*_S_ = 0.01 *P* = .98	*r*_S_ = −0.13 *P* = .25	*r*_S_ = −0.19 *P* = .11	*r*_S_ = −0.21 *P* = .07
Physical Condition	*r*_S_ = 0.03 *P* = .82	*r*_S_ = −0.24 ***P* = .04**	*r*_S_ = −0.23 ***P* = .047**	*r*_S_ = −0.24 ***P* = .04**
BES for males				
Physical Attractiveness	*r*_S_ = −0.20 *P* = .39	*r*_S_ = −0.13 *P* = .59	*r*_S_ = −0.31 *P* = .17	*r*_S_ = −0.18 *P* = .44
Upper Body Strength	*r*_S_ = −0.20 *P* = .40	*r*_S_ = −0.11 *P* = .62	*r*_S_ = −0.13 *P* = .59	*r*_S_ = −0.08 *P* = .72
Physical Condition	*r*_S_ = −0.22 *P* = .34	*r*_S_ = −0.17 *P* = .48	*r*_S_ = −0.09 *P* = .71	*r*_S_ = −0.08 *P* = .72

BES = Body Esteem Scale, CD = celiac disease, GFD = gluten-free diet, *r*_S_ = Spearman rank correlation coefficient, TAS-26 = Toronto Alexithymia Scale-26.

*P* values showing statistically significant difference are written in bold.

In the group of women, correlations were found between the impact of CD symptoms on hospitalizations and the BES subscales: sexual attractiveness (*r*_S_ = −0.31), weight concern (*r*_S_ = −0.23) and physical condition (*r*_S_ = −0.41).

Regarding the relationship between variables concerning the psychosocial aspects of dieting and psychological variables, correlations were found between the TAS-26 subscale: identification of feelings and statements regarding the impact of GFD on family life (*r*_S_ = 0.22). In the group of CD women, there was also a correlation between the BES domain, physical condition, and statements defining the degree of negative impact of GFD on family life (*r*_S_ = −0.24), professional life (*r*_S_ = −0.23) and emotional burden (*r*_S_ = −0.24).

### 3.5. Relationships between TAS-26 and BES in CD patients

Analysis of relations between alexithymia and body image in women with CD revealed weak associations between perception of physical condition and reduced daydreaming (*r*_S_ = −0.27) and total score of TAS-26 (*r*_S_ = −0.23). Interestingly, in the male sample, difficulty in identifying feelings is moderately and adversly related to physical attractiveness (*r*_S_ = −0.51), upper body strength (*r*_S_ = −0.44) and physical condition (*r*_S_ = −0.63). Moreover, physical condition is related to externally oriented thinking (*r*_S_ = −0.47) and to total score of TAS-26 (*r*_S_ = −0.49) (for details see Table 10).

## 4. Discussion

This study investigated the frequency and severity of alexithymia and body image disturbances in CD patients. To the best of our knowledge, this is the first report to explore these psychosocial constructs together in CD, highlighting a novel clinical dimension of the disease with potential implications for patient care.

### 4.1. Alexithymia in patients with CD

In this study, an attempt was made to investigate and characterize emotion regulation in 96 patients treated due to CD. In the available literature, there are papers on coexisting mood disorders in CD, but only a few concern the increased risk of alexithymia among these patients.^[9,10]^ Collin et al^[9]^ did not confirm the relationship between an increased level of alexithymia and CD as well as adherence to a GFD, however, Kreitler and Kreitler^[10]^ indicated a higher level of alexithymia in the group of patients with CD compared to the healthy controls. In addition, recognize of alexithymia increases the risk of depressive disorders, which in turn, as already indicated, are frequent comorbidities with CD.^[8]^

Studies conducted by Taycan et al^[22]^ unequivocally link alexithymia to an increased risk of diagnosis of somatic diseases. The authors indicate: a decrease in cellular immunity (lower production of specific cytokines) and an associated susceptibility to infections, a risk of hypertension, eating disorders, sleep disorders, depression, bronchial asthma, and dermatological problems.^[23]^ Interestingly, Guénolé et al^[24]^ indicated that alexithymia can be common in adolescents and young adults with severe idiopathic scoliosis, as in other diseases with bone structure disorders that can lead to pathological fractures.^[25–28]^ Considering the state-trait concept of alexithymia, it was proposed that alexithymia in idiopathic scoliosis might be recognized as a state-dependent condition (secondary alexithymia) which, contrary to primary alexithymia, is not constitutive of personality but rather reflects a repressive coping style toward illness-related affective adversity.^[24,29]^

Research results concerning the relationship between alexithymia and CD remain ambiguous.^[9,10]^ The results obtained in this study using TAS-26 only coincide in some areas with the results of studies by other authors. We indicated alexithymia in as many as 26% of patients with CD and in 8% of healthy controls. Interesting relationships were also found between deficits in the area of feelings identification and the intensification of the negative impact of GFD on family life. When interpreting these relationships, the correlational nature of the obtained results should be taken into account. On the one hand, these results may indicate the difficulties of people with deficits in emotional awareness with accepting the limitations resulting from following a GFD and functioning in various areas of family life. On the other hand, the confirmed correlations point to the importance of GFD restrictions that negatively affect family interactions and thus increase the level of emotional tension in relation to the efficient and adequate recognition of these emotions.

### 4.2. Body image in patients with CD

In 30 to 40% of adults with CD is reported the typical form of the disease, which includes diarrhea, abdominal pain, weight loss and nausea.^[6]^ As indicated by Pietras-Mrozicka,^[30]^ physiological disorders in patients with CD may significantly affect the assessment of their own body image, and body dissatisfaction may increase along with worsening symptoms of CD and weight loss. Due to the emerging characteristic clinical symptoms of CD, which negatively affect the physical appearance of patients, a psychometric tool assessing body image disorders (BES) was applied in this study.

Interestingly, in this study, there were no statistically significant differences in the examined BES subscales between women with CD and healthy controls. Among women, however, there were correlations between the perception of the impact of CD symptoms on hospitalizations and the perception of physical attractiveness, weight concern and physical condition. Significant correlations were also observed in this group between the perception of physical condition and the perception of the negative impact of dietary requirements on family and professional life as well as emotional burden. In the case of the BES subscales for men, significant differences, to the disadvantage of patients with CD, occurred in the perception of all assessed dimensions: physical attractiveness, body strength and physical condition.

The core feature of alexithymia is the inability to identify and verbalize emotions, which significantly impairs emotional regulation.^[31]^ Importantly, alexithymia may also be linked to disturbances in body image. Previous research by Mloźniak et al^[31]^ demonstrated that young adults with alexithymia perceive their bodies more negatively than those without alexithymia, reflecting difficulties in describing both emotions and attitudes toward their own body.

The present study confirms these findings in patients with CD. In women with CD, poorer perception of physical condition (such as stamina, strength, and agility) was associated with higher overall alexithymia and reduced daydreaming. In men, negative evaluations of endurance, strength, and agility were linked to difficulty identifying feelings and externally oriented thinking. These results highlight a clear association between body image disturbances and emotional regulation deficits in CD patients. Clinically, this suggests that assessment of emotional awareness and body image should be integrated into the routine evaluation of patients with CD, as addressing these psychosocial factors may support adherence to a GFD and improve their overall quality of life.

### 4.3. Study limitations

One of the main limitations of the study is the significant predominance of women in both groups studied. However, it is difficult to determine whether the demographic composition of the research group resulted from the greater awareness of women with regard to the diagnosis of the disease, or more frequent consent to participate in the study itself. This imbalance may limit the generalizability of the results to the broader population of patients with CD. Also, the cross-sectional and correlational design of this study limits conclusions to associations and prevents inferences about causality.

### 4.4. Future research implications

It would be valuable to conduct longitudinal studies assessing the emotional and cognitive functions of CD patients before and after achieving remission. Similarly, comparative studies including patients who have received at least 1 year of psychological support versus those without such support could provide further insights. Additionally, examining cognitive and emotional function disorders in patients with non-celiac gluten sensitivity, and comparing these findings with results from patients diagnosed with CD, could yield important clinical implications. Future prospective studies could help clarify the temporal relationships between disease activity, psychological support, and changes in emotional and cognitive functioning, thereby providing stronger evidence for causality and informing tailored interventions.

### 4.5. Practical clinical implications

The increased risk of body image disturbances in men with CD (across physical attractiveness, perceived body strength, and assessment of physical condition) highlights the importance of incorporating counseling and psychological support into patient care. Additionally, difficulties in identifying and describing emotions should be considered when planning diagnosis and treatment strategies, as they may influence adherence to a GFD and overall well-being.

## 5. Conclusions

In conclusion, our study highlights the importance of a comprehensive evaluation of male and female patients with CD, taking into account comorbidities such as alexithymia and body image disturbances. The results indicate that men with CD exhibit higher levels of alexithymia and body image disturbances compared to healthy controls. Overall, CD patients show alexithymic traits, although these appear largely unrelated to sociodemographic, clinical, or GFD-related factors. Notably, difficulty in identifying feelings negatively impacts body perception in male patients. Further research, including more comprehensive psychiatric assessments and personality testing, is needed to clarify how instruments such as TAS-26 and BES can be applied in daily clinical practice to improve CD management.

## Author contributions

**Conceptualization:** Kinga Kot, Agnieszka Dobrowolska, Iwona Krela-Kaźmierczak, Ewa Misterska.

**Data curation:** Kinga Kot.

**Formal analysis:** Kinga Kot.

**Investigation:** Kinga Kot.

**Methodology:** Kinga Kot, Iwona Krela-Kaźmierczak, Ewa Misterska.

**Project administration:** Kinga Kot.

**Supervision:** Agnieszka Dobrowolska, Iwona Krela-Kaźmierczak, Maciej Głowacki, Ewa Misterska.

**Validation:** Kinga Kot, Maciej Głowacki, Ewa Misterska.

**Writing** – **original draft:** Ewa Misterska.

**Writing** – **review & editing:** Kinga Kot, Agnieszka Dobrowolska, Iwona Krela-Kaźmierczak.
